# Prevalence of the colistin resistance gene *MCR-1* in colistin-resistant *Klebsiella pneumoniae* in Egypt

**DOI:** 10.3934/microbiol.2023011

**Published:** 2023-03-01

**Authors:** Rania Abozahra, Amal Gaballah, Sarah M. Abdelhamid

**Affiliations:** Department of Microbiology and Immunology, Faculty of Pharmacy, Damanhour University, Damanhour, Egypt

**Keywords:** *Klebsiella pneumoniae*, colistin resistance, *mcr-1* gene, rapid polymyxin NP test, transferability, eugenol

## Abstract

*Klebsiella pneumoniae* is a nosocomial pathogen with high morbidity and mortality rates in hospitalized patients. The emergence of multidrug-resistant *K. pneumoniae* has become more challenging to treat, with the prevalence of colistin-resistance. Therefore, reliable methods for detecting colistin resistance are required. Many plants' essential oils have antimicrobial activity and have been used to combat multiple antibiotic resistances. This study aimed to investigate the characterization and prevalence of the colistin resistance gene *mcr-1* in *K. pneumoniae* in Egypt, evaluate rapid polymyxin NP test, determine the transferability of *mcr-1* gene, and study the synergistic activity of eugenol combined with colistin against *K. pneumoniae* isolates. Eighty-two *K. pneumonia* isolates were collected from different human samples, followed by antibiotic susceptibility testing, rapid polymyxin NP test, and detection of the *mcr-1* gene and its transfer frequency. Determination of the MICs of colistin alone and in combination with eugenol was performed, then *mcr-1* gene expression was determined in the presence of eugenol. Thirty-two isolates (39%) were colistin-resistant. Rapid polymyxin NP test failed to detect resistant isolates with MICs below 32 µg/mL. Detection of *mcr-1* gene was made in 27 (84%) of colistin resistant isolates. The rest isolates possess alteration in the *mgrB* gene which probably causes colistin resistance. The *mcr-1* gene was transferred by conjugation to other sensitive isolates. MIC of eugenol ranged from 416 to 1664 µg/mL, and FICI ranged from 0.265 to 0.75. Results also revealed suppression of *mcr-1* gene expression in the presence of sub MIC of eugenol. Our results demonstrated a high prevalence of *mcr-1* in Egypt and its ability to transfer to other strains. Difficult determination of colistin-resistant isolates with low values with rapid polymyxin NP test was apparent. Eugenol exerted a synergistic effect with colistin and improved its antimicrobial activity.

## 1. Introduction

*Klebsiella pneumoniae* is a Gram-negative, rod-shaped, non-motile, facultatively anaerobic, lactose-fermenting bacillus with a prominent capsule belonging to the family Enterobacteriaceae [1]. It is recognized as an opportunistic organism. It is the main cause of approximately 15% of all cases of community-acquired pneumonia in Africa and approximately 11.8% of all cases of hospital-acquired pneumonia in the world [2]. It is also one of the main sources of ventilator-related pneumonia (VAP) among patients in intensive care units (ICUs) [3], and causes 83% of hospital-acquired (HA) pneumonia [4]. Death rates in *K*. *pneumoniae* pneumonia have been accounted for as high as 50% [2].

The increase of multi-drug resistant (MDR) Gram-negative bacteria and the decrease in the discovery of new antibiotics have forced clinicians to reuse colistin as the last resort to overcome these superbugs [5]. Colistin or polymyxin E is a cationic antibiotic effective against Gram-negative bacteria [6]. Neurotoxicity and nephrotoxicity, side effects of colistin use, have [7] limited its utility in 1970 [8].

The cationic region of polymyxin interacts electrostatically with the negatively charged lipid A moieties of lipopolysaccharides (LPS) that are present on the outer membrane (OM) of Gram-negative bacteria. It replaces divalent cationic ions (e.g., Mg2+ and Ca2+), which affects the permeability of OM. This leads to cellular component leakage, followed by cell death [8],[9].

Unfortunately, colistin resistance has emerged [10]. Colistin resistance results from the modification of lipid A by the addition of phosphoethanolamine (pETN), and/or 4-amino-L-arabinose (L-Ara4N) that leads to an increase in its positive charges reducing its affinity to colistin [11]. It is caused either by chromosomal mutation of the two-component regulatory system of bacteria *PmrA/PmrB* and *PhoP-PhoQ* [12] and its negative feedback *mgrB* gene [13],[14] or by expression of plasmid-encoded *MCR* enzymes that spread worldwide [15] after the emergence of *mcr-1* in China for the first time in 2015 [16]. The presence of plasmid facilitates the dissemination of genes through the mechanism of horizontal gene transfer [17]. This is why finding effective combination therapy is crucial to get rid of colistin-resistant bacteria and slowing down its spread and prevalence.

Eugenol is the major active essential component of clove oil that is obtained naturally from Eugenia aromatica. It has analgesic, local anesthetic, and anti-inflammatory effects. It is used in the form of a paste or mixture as dental cement, filler, and restorative material [18]. Eugenol has antibacterial action; it affects membrane permeability and interacts with protein and enzymes inside the cell leading to its destruction [19]. It has an antimicrobial activity against *Escherichia coli*, *K. pneumoniae*, *Acinetobacter baumanni*, *Staphylococcus aureus*, and *Enterococcus faecalis*.it also exhibited the best antibacterial activity against *Streptococcus gordonii*, *Porphyromonas gingivalis* and *Streptococcus mutans* [20]. Furthermore, the synergistic combinations with other EOs and conventional antimicrobials have also been highly publicized since the last decade [21]. Eugenol is safe in low doses with a few side effects other than local irritation, rare allergic reactions, and contact dermatitis. Exposure or ingestion of large amounts, as in overdose, can result in tissue injury and a syndrome of acute onset of seizures, coma, and damage to the liver and kidneys [22].

To date, very few studies about colistin-resistant *K. pneumoniae* isolated from Egyptian patients are available So, our study aimed to investigate the characterization and prevalence of the colistin resistance gene *mcr-1* in *K. pneumoniae* collected from human clinical specimens in Egypt, evaluate rapid polymyxin NP test, determine the transferability of *mcr-1* gene, and study the synergistic activity of eugenol combined with colistin against a collection of clinical *K. pneumoniae* isolates.

## 2. Materials and methods

### 2.1. Sample collection and antibiotic susceptibility profiling

Eighty-two clinical isolates of *K. pneumoniae* were collected from the microbiology laboratory of Damanhour medical national institute and the Microbiology Department at El Mery Hospital. They were collected from different clinical specimens including blood, pus, sputum, urine, tracheal tube, and wound. The isolates were identified using biochemical testing and colistin-resistant isolates were confirmed by the Vitek system.

### 2.2. Disc diffusion method

The susceptibility of the clinical isolates to Gentamicin (GMN, 10 µg), Etrapemem (ETP: 10 µg), Aztreonam (ATM: 30 µg), Cefepime (CPM: 30 µg), Amikacin (AK: 30 µg), Tetracycline (TE: 30 µg), Ceftriaxone (CTR, 30 µg), Ciprofloxacin (CIP: 5 µg), Cefuroxime (CXM: 30 µg), Trimethoprim-sulfamethoxazole (COT: 1.25/23.75 µg), Ampicillin-sulbactam (SAM: 10/10 µg), Ampicillin (AMP: 10 µg), Piperacillin (PRL: 100 µg) was performed using the standard diffusion method according to Bauer *et al*. [23]with some modifications [24]. The diameter of each inhibition zone generated around the disc was measured in mm and compared to susceptibility tables of the Clinical and Laboratory Standards Institute (CLSI 2021) [25] to determine the susceptibility of isolates and interpretation of results either susceptible (S), intermediate (I) or resistant (R). The antibiotic disks used were purchased from Oxoid (Oxoid Ltd; Basingostok; Hampshire, England).

### 2.3. Determination of the minimum inhibitory concentration (MIC)

The broth microdilution method (BMD) was performed according to the EUCAST/CLSI guidelines to quantify antibacterial resistance against colistin. Different dilutions of colistin (ACROS organics; Belgium) ranging from 0.125 to 128 mg/mL were made in cation-adjusted MH broth (HiMedia Laboratories Pvt., Mumbai, India) and inoculated with the tested organism giving a final concentration of 5 × 10^5^ CFU/mL of bacteria in each well. This procedure was performed in triplicate for each tested organism. The bacterial cultures were incubated at 37 °C for 18–20 h and then visually examined for microbial growth to determine the MIC values as the lowest concentration of the antibiotic that inhibited the growth of the microorganism. The reference breakpoint for the interpretation of MIC against colistin was set as mentioned by CLSI 2021, a MIC ≤ 2 µg/mL was intermediate, and ≥4 µg/mL was categorized resistant [26],[27].

### 2.4. Rapid polymyxin NP test

The Rapid polymyxin NP test is based on the detection of bacterial metabolism in the presence of a 3.75 µg/mL colistin concentration in a cation-adjusted Müller-Hinton broth (MH) medium. The change in color of phenol red (pH indicator) from orange/red to yellow after incubation at 35 °C ± 2 °C for 2 hours indicates colistin resistance as it grows and forms acid metabolites consecutive to the glucose metabolism. Colistin-susceptible and colistin-resistant reference bacterial suspensions were used as a negative and positive control, respectively [28],[29].

### 2.5. Induction of colistin resistance among selected sensitive isolates

100 µL of an overnight culture of thirty sensitive clinical isolates were subcultured in 100 µL cation-adjusted MH broth containing 1/4 MIC of colistin for five consecutive days to induce colistin resistance. MIC value of the induced isolates to different concentrations of colistin was measured by the BMD method [30].

### 2.6. PCR assay

The primers for *mcr-1* and its amplicon size are listed in (Table 1). Plasmid DNA templates were obtained by using a QIAprep® Spin Miniprep kit (Qiagen, Hilden, Germany) to one colony grown overnight on LB medium.

*pmrAB* and *phoPQ* and its negative regulator *mgrB* were screened and amplified using the primers described in table 1. The DNA was isolated with a QIAamp® DNA Mini kit (Qiagen, Hilden, Germany).

Each PCR reaction consisted of 12.5 µL of My Taq™ HS Red Mix PCR master mix (Bioline, UK), 1 µL DNA extract, 1 µL forward primer (10 pmol/µL), 1 µL reverse primer (10 pmol/µL) and 9.5 µL water.

The PCR cycling condition was as follows: 1 cycle of denaturation at 95 °C for 1 min, 35 cycles of denaturation at 95 °C for 15 seconds followed by annealing at 55 °C for 15 seconds and elongation at 72 °C for 10 seconds, and final elongation cycle at 72 °C for 10 minutes. The PCR products were loaded on a 2% agarose gel containing ethidium-bromide and visualized after 30 minutes of electrophoresis at 120 V.

**Table 1.** The sequences of the primers used in the study.

Primer name	Neucleotide sequence (5′–3)′	No. of bases	Size of the amplicons (bps)	Reference
*mcr-1*-F	AGTCCGTTTGTTCTTGTGGC	20	320	[31]
*mcr-1*-R	AGATCCTTGGTCTCGGCTTG	20		[31]
*phoP*-F	ATTGAAGAGGTTGCCGCCCGC	21	136	[13]
*phoP*-R	GCTTGATCGGCTGGTCATTCACC	23		[13]
*phoQ*-F	CTCAAGCGCAGCTATATGGT	20	177	[11]
*phoQ*-R	TCTTTGGCCAGCGACTCAAT	20		[11]
*pmrA*-F	GATGAAGACGGGCTGCATTT	20	104	[11]
*pmrA*-R	ACCGCTAATGCGATCCTCAA	20		[11]
*pmrB*-F	TGCCAGCTGATAAGCGTCTT	20	94	[11]
*pmrB*-R	TTCTGGTTGTTGTGCCCTTC	20		[11]
*mgrB*-F	CGGTGGGTTTTACTGATAGTCA	22	110	[14]
*mgrB*-R	ATAGTGCAAATGCCGCTGA	19		[14]

### 2.7. DNA sequencing

Full nucleotide sequences of *mgrB* of four isolates that did not possess the *mcr-1* gene were determined by direct DNA sequencing using primers listed in Table 1. Gene JET PCR Purification Kit (Thermo Scientific, K0701) was used for DNA purification after its amplification. ABI PRISM® 3100 Genetic Analyzer was applied for sequencing PCR products performed by Macrogen In. Seal, Korea. Gel documentation system (Geldoc-it, UVP, England), was applied for data analysis using Totallab analysis software, ww.totallab.com, (Ver.1.0.1). Aligned sequences were analyzed on the NCBI website (http://www.ncbi.nlm.nih.gov/webcite) using BLAST to confirm their identity. The Genetic distances and MultiAlignments were computed by the Pairwise Distance method using ClusteralW software analysis (www.ClusteralW.com). Nucleotide sequences were also compared with bacterial isolate sequences available in the GenBank.

Nucleotide sequence accession numbers. The nucleotide sequences of the altered *mgrB* genes have been deposited at GenBank under the accession numbers LC720456 (isolate AG001), LC720457 (isolate AG002), LC720458 (isolate AG003), and LC720459 (isolate AG004).

### 2.8. Transferability of plasmid by conjugation

Colistin-resistant isolates of the *K. pneumoniae* were selected as the plasmid donor and mixed with the recipient (colistin-sensitive isolates) in a ratio of 2:1. They were spotted onto Luria-Bertani agar (LB agar) at 28 °C for 16 h after its harvest by centrifugation. Then 5 mL of LB broth was used to resuspend the cells followed by its culture onto non-selective and selective LB agar containing both 4 µg/mL colistin and 64 µg/mL amikacin and incubated at 37 °C [32]. The grown colonies were diluted and counted to determine the transfer frequencies by dividing the number of transconjugants by the number of donor cells [33].

### 2.9. Plasmid transformation into E. coli and K. pneumoniae strains

Plasmid extract selected from two different colistin-resistant isolates was transformed by heat shock technique into chemically competent *E. coli* DH5α and sensitive-isolate *K. pneumoniae*. The chemically competent cells were prepared by using CaCl_2_ and MgSO_4_ solutions after their sterilization by autoclaving at 121 °C for 15 min. The cells were mixed with 5 µL of plasmid extract and incubated on ice for 15 min followed by exposure to heat and transferred back on ice. Bacterial cells were regenerated by adding LB broth and incubation for 1 h then plated on selective LB agar containing 4 µg/mL colistin. The plates were checked for any transformants after its incubation for 24 h at 37 °C [34].

### 2.10. Checkerboard assay

The antibacterial effects of the combination of eugenol and colistin were evaluated by checkerboard test as described in previous studies [35]. The concentrations tested of each agent usually ranged from 4 times below the MIC to 2 times the MIC using the two-fold serial dilution method (i.e., from ^1^/_16_ to double the MIC).

The MICs of the individual drugs, colistin, eugenol, and the combinations, were determined using the broth microdilution technique as recommended by the CLSI and described above. Each longitudinal column tube contained the same concentration of drug A and each horizontal row of tubes contained the same concentration of drug B. All tubes were inoculated with bacterial suspension giving a final concentration of approximately 5 × 10^5^ CFU/mL. Colistin-free control tubes, eugenol-free control tubes, and blank control tubes were also set, and all tubes were incubated at 37 °C for 16 h under aerobic conditions. The experiment was triplicated.

After the determination of the MICs of single drugs A and B (MIC_A_ and MIC_B_) and in combination (MIC_AB_ and MIC_BA_), the Fractional Inhibitory Concentration (FIC) index was calculated to deduce the antibacterial activity of each combination by using the formula:

FICI=FICA+FICB

where FICA equals the MIC of drug A in combination is divided by the MIC of drug A alone. FICB is the same as FICA but for drug B, the MIC of drug B in combination divided by the MIC of drug B alone.

Combination efficacy should be determined as follows:

Synergism was defined when FICI ≤ 0.5 while 0.5 < FICI ≤ 0.75 indicated partial synergy. Additivity was donated by 0.76 < FICI ≤ 1 while 1 < FICI ≤4 denoted Indifferently and antagonism in cases in which the FIC index > 4.

### 2.11. Real-time PCR

The effect of eugenol on the expression of the *mcr-1* gene at the mRNA level was estimated using Real-time PCR. Eugenol was mixed with bacterial cultures in the logarithmic phase to make a final concentration of sub-MIC (3/4 MIC). While LB broth was added instead of eugenol in the control group. The cells were grown at 37 °C with shaking at 160 rpm for 16 h. Total RNA was isolated using an RNeasy® Mini Kit (Qiagen, Hilden, Germany) according to the manufacturer's instructions.

Real-time PCR performed in an Applied Biosystems step one Real-Time PCR System (USA) using TOPreal™ One-step RT qPCR Kit (SYBER Green with low ROX) (enzynomics, Daejeon, South Korea). Each PCR reaction tube contained 20 µL reaction mixtures consisting of the following:1 µL TOPreal ™ One-step RT qPCR Enzyme Mix, 10 µL TOPreal™ One-step RT qPCR Reaction Mix, 2 µL RNA extract, 1 µL of each primer and 5 µL RNAase free water. *GapA* [36] *and rpoB* [37]were used as reference genes to normalize expression levels.

The reacting condition was set as one step method as follows: synthesize cDNA at 42 °C for 30 min, initial denaturation at 94 °C for 3 min, 30 cycles consisting of denaturation at 94 °C for 30 sec, annealing at 55 °C for 30 sec. Data were calculated using the Comparative CT method and expressed as the mean ± standard deviation.

## 3. Results

### 3.1. Antimicrobial susceptibility test

**Figure 1.**
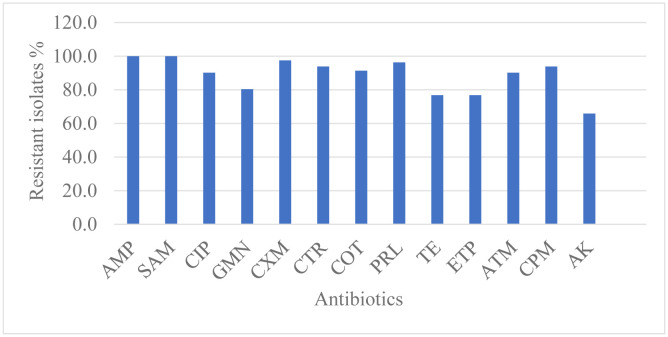
Resistance of *K. pneumoniae* isolates to the tested antibiotics.

Among the 82 *K. pneumoniae* isolates analyzed, 53.6% were resistant to all antibiotics except colistin. The rate of antibiotic resistance among the tested isolates was studied and the percentage of resistant isolates towards each tested antibiotic is shown in Figure 1.

### 3.2. Broth microdilution method

Thirty-two isolates were colistin-resistant, and fifty isolates were defined as colistin-intermediate according to the MIC breakpoints of colistin (CLSI 2021).

### 3.3. Rapid polymyxin NP test

Upon comparing the results of BMD method and polymyxin NP test, it was found that fifteen isolates defined as colistin-resistant according to the results of the BMD method were found susceptible by rapid polymyxin NP test as shown in Table 2.

**Table 2.** The MICs of colistin-resistant *K. pneumoniae* with performance evaluation of the rapid polymyxin NP test and its mechanism of resistance.

MIC (µg/mL)		No. of isolates (n = 82)	Isolate code	Mechanism of resistance	Rapid polymyxin NP test	CA*
Resistant isolates	128	4	K14	*mcr-1* positive	R*	100%
			K22	*mcr-1* positive	R*	
			K43	*mcr-1* positive	R*	
			K44	*mcr-1* positive	R*	
	64	2	K11	*mcr-1* positive	R*	100%
			K13	*mcr-1* positive	R*	
	32	3	K9	*mcr-1* positive	R*	100%
			AG003	*mcr-1* positive	R*	
			K55	*mcr-1* positive	R*	
	16	6	K1	*mcr-1* positive	R*	83.3%
			K5	*mcr-1* positive	R*	
			AG001	Chromosomal encoded	S*	
			K8	*mcr-1* positive	R*	
			AG002	Chromosomal encoded	R*	
			K31	*mcr-1* positive	R*	
	8	7	K2	*mcr-1* positive	S*	14.3%
			K3	*mcr-1* positive	R*	
			K4	*mcr-1* positive	R*	
			K18	*mcr-1* positive	S*	
			K64	*mcr-1* positive	R*	
			K61	*mcr-1* positive	S*	
			AG004	Chromosomal encoded	S*	
	4	10	K17	*mcr-1* positive	S*	0
			K23	*mcr-1* positive	S*	
			K62	*mcr-1* positive	S*	
			K36	*mcr-1* positive	S*	
			K39	*mcr-1*positive	S*	
			K67	*mcr-1* positive	S*	
			K63	*mcr-1* positive	S*	
			K65	*mcr-1* positive	S*	
			K77	*mcr-1* positive	S*	
			K71	Chromosomal encoded	S*	
Susceptible isolates	2	19			S*	100%
	1	22			S*	100%
	0.5	9			S*	100%

No. of ME = 0

No. of VME = 15 (45.5%)

S*, colistin-susceptible; R*, colistin-resistant; CA*, categorical agreement; ME, major errors; VME, very major errors. The data represents low-level resistance isolates (MICs 4 or 8 µg/mL), where the lowest categorical agreement was observed.

### 3.4. Induction of colistin resistance among selected sensitive isolates

**Figure 2.**
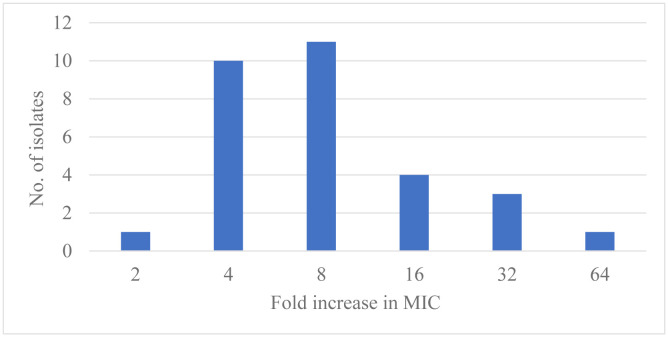
Induction of resistance in the selected isolates by using ¼ MIC of colistin.

After five passages of thirty isolates in ¼ MIC of colistin, the MIC of 3.3 %, 33.3 %, 36.7%, 13.3%, 10%, and 3.3% of isolates increased by two-fold, four-fold, eight folds, 16 folds, 32 folds, and 64 folds, respectively (Figure 2).

### 3.5. PCR assay and sequencing

The PCR protocol specifically amplified the fragments of the *mcr-1* gene with 320 bp amplicon size It was found that twenty-seven isolates out of thirty-two harbor the *mcr-1* gene that causes resistance to colistin. *phoP, phoQ, pmrA, pmrB, and mgrB* genes were detected in all isolates.

**Figure 3.**
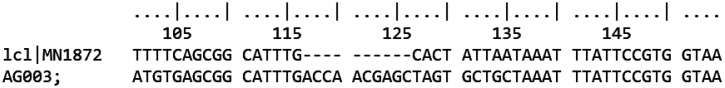
Sequence of the *mgrB* gene in isolate AG003 indicates the target site for insertion.

The occurrence of *mgrB* alterations in colistin-resistant clinical isolates of AG001, AG002, AG003, and AG004 was detected by PCR mapping and sequencing strategy using primers described in Table 1. The amplification products found in the four isolates were inactivated by point mutation and small or even large deletion of nucleotides. The gene sequencing of the *mgrB* gene in isolate AG003 was modified by the insertion of additional nucleotides between nucleotide positions 116 and 126 as shown in Figure 3. The four isolates possess a premature stop codon leading to truncated proteins. All these alterations caused the substitution of amino acids that were probably leading to a non-functional MgrB protein, and thus were possibly the source of colistin resistance.

### 3.6. Transferability of plasmid by conjugation

**Figure 4.**
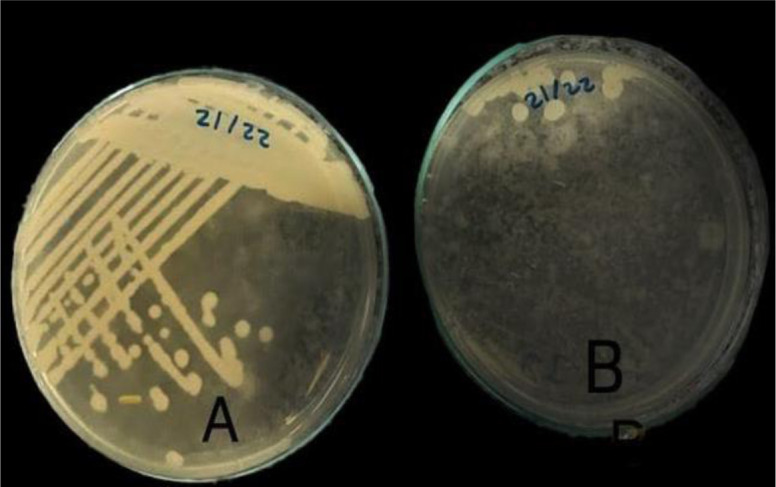
A: recipient in presence of colistin and amikacin; B: recipient in presence of colistin.

Conjugation between three *K. pneumoniae* clinical isolates harboring the *mcr-1* gene as a donor and colistin-sensitive *K. pneumoniae* isolates as a recipient was done to check the potential transfer of plasmid-mediated colistin resistance horizontally. After selection, variable numbers of transformants were observed (Figure 4). Their MIC values and transfer frequencies were calculated as shown in Table 3.

**Table 3.** Plasmid transfer frequencies and MIC of colistin against obtained transformants and recipient isolate.

Isolate code	Transfer frequency	MIC of colistin (µg/mL) against	
		recipient isolate	obtained transformants
K22	2.4 × 10^-5^	1	128
K77	6.1 × 10^-6^	1	4
K3	2.6 × 10^-5^	2	8

### 3.7. Plasmid transformation into E. coli and K. pneumoniae strains

Plasmids harboring *mcr-1* from *K. pneumoniae* isolate detected by PCR were successfully transformed in *E. coli* DH5α and sensitive strain *K. pneumoniae* using a heat shock technique. They grew in LB agar containing colistin and their MIC was 4 µg/L which confirms the functionality of the gene.

### 3.8. Synergistic effect of eugenol with colistin

The combination of colistin with eugenol was tested against 27 selected clinical isolates. The results of the antimicrobial activity showed that eugenol presented variable antimicrobial activity against all tested strains (MIC, 416 to 1664 µg/mL), and the FIC indices obtained ranged from 0.265–0.75. The obtained results showed that the combination was synergistic against 37.03%, and partial synergistic against 62.96%, as shown in Table 4.

**Table 4.** MIC and FICI of eugenol and colistin against *K. pneumoniae* isolates.

Isolate code	MIC colistin		MIC eugenol		FICI index	Combination efficacy
	Alone	In combination	Alone	In combination		
K1	16	2	832	416	0.625	Partial synergy
K2	8	1	832	416	0.625	Partial synergy
K3	8	1	1664	832	0.625	Partial synergy
K4	8	2	1664	416	0.5	Synergy
K5	16	2	832	208	0.37	Synergy
K8	16	2	1664	832	0.625	Partial synergy
K9	32	1	832	416	0.53	Partial synergy
K11	64	2	832	416	0.531	Partial synergy
K13	64	4	416	208	0.65	Partial synergy
K14	128	4	832	416	0.53	Partial synergy
K17	4	2	832	208	0.75	Partial synergy
K18	8	1	832	416	0.625	Partial synergy
K22	128	2	832	416	0.5	Synergy
K23	4	2	832	104	0.625	Partial synergy
K31	16	1	1664	832	0.56	Partial synergy
K36	4	2	832	104	0.625	Partial synergy
K39	4	2	832	104	0.625	Partial synergy
K43	128	2	832	208	0.265	Synergy
K44	128	8	832	208	0.312	Synergy
K55	32	4	823	208	0.375	Synergy
K61	8	2	832	104	0.375	Synergy
K62	4	1	832	208	0.5	Synergy
K63	4	1	832	416	0.75	Partial synergy
K64	8	2	832	208	0.5	Synergy
K65	4	1	1664	416	0.5	Synergy
K74	4	2	832	104	0.625	Partial synergy
K77	4	2	1664	416	0.75	Partial synergy

### 3.9. Real-time PCR

**Figure 5.**
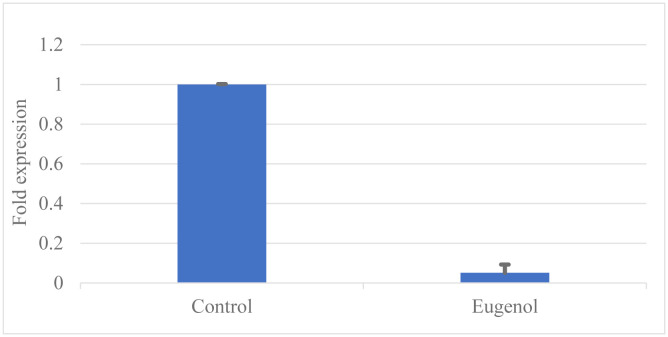
Expression of *mcr-1* gene in the presence and absence of 3/4MIC of eugenol. Data were expressed as mean ± S.D.

The expressions of *mcr-1* were compared before and after the addition of eugenol to investigate if eugenol influences drug-resistant genes at the mRNA level. They were assessed using qPCR and using two housekeeping genes *rpoB* and *gapA* as reference standards. The addition of 3/4MIC of eugenol was able to decrease the expression of the *mcr-1* gene in *K. pneumoniae* isolates. The results in Figure 5 presented differences in the *mcr-1* gene before and after eugenol addition, which indicates that colistin resistance gene *mcr-1* was down-regulated by additional eugenol when compared to untreated *K. pneumonia*.

## 4. Discussion

*Klebsiella pneumoniae* is classified as one of the most serious ESKAPE organisms that effectively escape antibacterial drugs [38], even when colistin was the last choice for its treatment [39]. In the present study, the susceptibility test displayed that 53.6% of our isolates were resistant to all antibiotics used. It was found that 76.8–100% of tested isolates were resistant to β-lactam antibiotics. These results were in accordance with the results of Montso et al. where 66.7–100% of their isolates were resistant to *β*-lactams [40]. Moreover, our tested isolates showed 76.8% resistance to ertapenem, while a study by Oladipo et al. showed 91% of *K. pneumoniae* isolates were highly susceptible to ertapenem [41]. In contrast to a study by Kareem et al. where resistance for amikacin and gentamicin were 48.8% and 69.7%, respectively [42], our study showed high resistance to aminoglycosides where the resistance ranged from 65.9–80.4%. High resistance was observed towards ciprofloxacin at 90.2% which was in accordance with Karimi et al. who found resistance to be 80% [43].

MIC values of colistin ranged from <0.5–128 µg/mL. Thirty-two (39%) isolates were resistant to colistin in our study. Zafer et al. reported 22 (4.9%) colistin-resistant *K. pneumoniae* isolated over 18 months from cancer patients in Egypt [44]. Colistin resistance was detected in 8 (7.5%) *K. pneumoniae* in Tanta University Hospitals according to Ezzat et al. [45]. Rabie et al. found that 17.2% of *K. pneumoniae* isolates were resistant to colistin at Zagazig University Hospitals [46]. The variability in susceptibility of *K. pneumoniae* isolates toward colistin in different studies from Egypt may be due to differences in geographical zones or using different protocols of antibiotics in these regions.

Rapid polymyxin NP Test is a new phenotypic test which has been developed and evaluated worldwide to detect colistin resistance. Our study confirms that it was simple, easy to perform, and fast, as determined in other studies [47]–[49]. The specificity of the rapid polymyxin NP test was found to be 100% which was similar to the studies of [49]–[51], Dalmolin et al. (98%), Nordmann P et al. (95.4%) and Conceição-Neto et al. (94%) [28],[47],[48], though slightly different from the results of Malli E et al. (82%) [52]. The sensitivity of the rapid polymyxin NP test was 53.1% in our study which was different from other international studies poirel et al. (100%), Nordmann P et al. (99.3%), Malli E et al (99%), Dalmolin et al. (98%), Shoaib et al. (97.2%) [28],[47],[49],[51],[52]. The study performed by Simar S et al. showed low sensitivity 25% [50] which could be due to possible heteroresistant isolates in their study [51]. In our study, the MIC of 14 isolates out of 15 colistin-resistant isolates that showed false negative results in rapid polymyxin NP test were close to the breakpoint (4 and 8 µg/mL), which was comparable to Conceição-Neto et al study that emphasizes the difficulty of detecting resistance in isolates with low MICs [48].

Regarding the study of the capability of colistin to prompt resistance against itself, it was found that colistin could induce resistance towards itself in all the selected isolates proved by increasing their MIC values by many folds ranging from 2–64 folds. This can be explained by mutations in the *pmrAB* and *lpxACD* genes [53], which have a role in colistin resistance. Also, mutations in several other genes were additionally observed (e.g., *vacJ* and *pldA*) that linked with the target of polymyxins; the outer membrane may be the reason [30].

Contrary to *mcr* gene families (*mcr-2* to *mcr-5*), The *mcr-1* gene is the most commonly to cause colistin resistance in humans [54], so we focused on it in our study. According to the results of PCR, 27 of the resistant studied isolates (84.4%) were positive for the *mcr-1* gene declared high prevalence rate of *mcr-1* in Alexandria and El-Beheira, Egypt. Previous studies detected a low prevalence of *mcr*-1 positive isolates from human clinical samples [44],[46],[55]. The higher rates of *mcr-1* carriage may be due to the high amount of livestock and poultry in El-Beheira.

*MgrB* is a small regulatory transmembrane protein. It is produced after activation of the *PhoQ/PhoP* signaling system and exerts negative feedback on the same system. Inactivation of the *mgrB* gene in *K. pneumoniae* leads to the upregulation of signaling systems which modify the LPS by adding 4-amino-4-deoxy-L-arabinose to lipid A, which decreases its affinity to polymyxins [13],[14]. In this work, we found that the four isolates which didn't have the *mcr-1* gene carried alterations of the *mgrB* gene that were possibly responsible for their colistin resistance. Several different genetic alterations were observed such as point mutations, small or even large deletions, and insertional inactivation that reflect several independent mutational events of *mgrB*. Our findings agree with Cannatelli et al. [14] study that found the same observation in addition to the inactivation of *mgrB* by IS*5*-like elements, which was the most common mechanism of *mgrB* alteration.

The spread of the *mcr-1* gene between *K. pneumoniae* and *E. coli* may generate pan-drug-resistant isolates such as those producing *mcr-1* and carbapenemases, therefore it is important to recognize and monitor its transfer [56]. Plasmid-mediated transfer of the *mcr-1* gene was investigated in our study through conjugation and heat shock transformation. Successful conjugative transfers were obtained between *K. pneumoniae* isolates in agreement with Dénervaud Tendon et al. [57]. Also, transformants were obtained by heat shock technique when *E. coli* DH5α and *K. pneumoniae* were used as recipients. These findings were in agreement with Ovejero et al. [58] and Zurfluh et al. [59].

The dissemination and high transfer rate of the *mcr-1* gene make it necessary to search for alternatives to substitute antibiotics. Essential oils (Eos) were proved to be one of these alternatives that have significant antimicrobial activity against a wide range of microorganisms [60]. Eugenol, the principal chemical component of clove oil is recognized as a safe compound with a recommended dose of 2.5 mg/kg body weight for humans according to Food and Agriculture Organization/WHO Expert Committee on Food Additives [61]. It has antibacterial activity against *K. pneumoniae* [62]. In the present study, Eugenol showed MIC of 416–1664 µg/mL among *K. pneumoniae* isolates. In Dhara et al. study, the MIC value of eugenol was 63–999 µg/mL [62]. Eugenol exhibited a synergistic effect on 11 out of 27 *mcr*-1 colistin-resistant isolates (FICI, 0.265 to 0.5), and a partial synergistic effect (FICI, 0.53–0.75) for the rest isolates. In general, the presence of eugenol decreased the MIC of colistin by 2 to 64-fold.

Real-time PCR results revealed that eugenol inhibits *mcr-1* gene expression as the expression of the *mcr-1* gene in the synergy group was significantly lower than in the nonsynergy group. This result is in accordance with Wang et al study [63], which suggests the synergistic effect is due to the interactions between the phenolic hydroxyl group of eugenol and MCR-1 protein.

## 5. Conclusion

In conclusion, there is a high prevalence of *mcr*-1 in Egypt due to its ability to transfer to other strains. Detection of colistin-resistant isolates with low values is difficult to be determined by rapid polymyxin NP test. Eugenol can promote health and reduce antibiotic resistance as it exerts a synergistic effect with colistin and improves its antimicrobial activity.
